# The Relationship Between Electronic Health Record System and Performance on Quality Measures in the American College of Rheumatology’s Rheumatology Informatics System for Effectiveness (RISE) Registry: Observational Study

**DOI:** 10.2196/31186

**Published:** 2021-11-12

**Authors:** Nevin Hammam, Zara Izadi, Jing Li, Michael Evans, Julia Kay, Stephen Shiboski, Gabriela Schmajuk, Jinoos Yazdany

**Affiliations:** 1 Division of Rheumatology Department of Medicine University of California San Francisco, CA United States; 2 Department of Epidemiology and Biostatistics University of California San Francisco, CA United States; 3 Philip R Lee Institute for Health Policy Research San Francisco, CA United States; 4 San Francisco Veterans Affairs Medical Center San Francisco, CA United States

**Keywords:** rheumatoid arthritis, electronic health record, patient-reported outcomes, quality measures, electronic health record, disease activity, quality of care, performance reporting, medical informatics, clinical informatics

## Abstract

**Background:**

Routine collection of disease activity (DA) and patient-reported outcomes (PROs) in rheumatoid arthritis (RA) are nationally endorsed quality measures and critical components of a treat-to-target approach. However, little is known about the role electronic health record (EHR) systems play in facilitating performance on these measures.

**Objective:**

Using the American College Rheumatology’s (ACR’s) RISE registry, we analyzed the relationship between EHR system and performance on DA and functional status (FS) quality measures.

**Methods:**

We analyzed data collected in 2018 from practices enrolled in RISE. We assessed practice-level performance on quality measures that require DA and FS documentation. Multivariable linear regression and zero-inflated negative binomial models were used to examine the independent effect of EHR system on practice-level quality measure performance, adjusting for practice characteristics and patient case-mix.

**Results:**

In total, 220 included practices cared for 314,793 patients with RA. NextGen was the most commonly used EHR system (34.1%). We found wide variation in performance on DA and FS quality measures by EHR system (median 30.1, IQR 0-74.8, and median 9.0, IQR 0-74.2), respectively). Even after adjustment, NextGen practices performed significantly better than Allscripts on the DA measure (51.4% vs 5.0%; *P*<.05) and significantly better than eClinicalWorks and eMDs on the FS measure (49.3% vs 29.0% and 10.9%; *P*<.05).

**Conclusions:**

Performance on national RA quality measures was associated with the EHR system, even after adjusting for practice and patient characteristics. These findings suggest that future efforts to improve quality of care in RA should focus not only on provider performance reporting but also on developing and implementing rheumatology-specific standards across EHRs.

## Introduction

The routine collection of disease activity (DA) and patient-reported outcomes (PROs) such as functional status (FS) in rheumatoid arthritis (RA) are nationally endorsed quality measures and an important component of tracking outcomes and improving care [1-3]. The process of collecting these assessments is essential to the implementation of a treat-to-target strategy, which has been shown to improve outcomes for patients with RA and decrease health care utilization [4]. Nevertheless, studies have identified that quality of care provided to patients with RA remains inconsistent [5,6].

Data from the early years of the American College of Rheumatology’s (ACR’s) national Rheumatology Informatics System for Effectiveness (RISE) Registry, an electronic health record (EHR)–enabled registry, showed that among more than 150,000 patients with RA, only 50% had an RA DA score or a FS recorded as structured EHR data. The use of structured data fields facilitates disease monitoring, data retrieval, and quality reporting, especially in comparison to storing information in free text fields (ie, clinical notes) alone [7]. Several groups have developed EHR-based applications to help support the documentation of DA and FS measures as structured fields [8-11], and some EHR vendors have incorporated similar tools into their foundation software. However, no studies have evaluated the relationship between the EHR system and performance on important quality measures on a national scale across rheumatology practices.

In this study, we use the ACR’s RISE registry to analyze the relationship between major EHR systems used by US rheumatologists and performance on DA and FS quality measures. In addition, we report on the characteristics of EHRs with higher performance on these measures.

## Methods

### Data Source

RISE is a national registry that passively collects data from EHRs of participating practices, aggregates and centrally analyzes performance on quality measures in rheumatology [12]. RISE practices have an on-site “registry connector” that uploads EHR data to the RISE clinical data warehouse on a nightly basis. Data that are uploaded include information regarding patient diagnoses, medications, laboratory studies, and vital signs.

When a practice first joins RISE, practice personnel work with registry staff to map structured data elements to relevant quality measures. Occasionally, practices request data elements to be pulled from clinical notes. This is feasible when data are recorded in a highly reliable, “semi-structured” format. Data elements extracted from the EHR are used to calculate electronic quality measures for submission to Centers for Medicare & Medicaid Services (CMS) as part of national pay-for-performance programs. For example, patients with RA are identified using ICD codes to enter a denominator population. DA scores are extracted (usually from structured fields) to determine whether denominator patients meet the criteria established by quality measures. Practice, provider, and patient-level performance on quality measures is fed back to providers through a web-based dashboard. Patient-level EHR data that include all variables mentioned above, in addition to quality measure denominator and numerator information is provided to RISE Data Analytic Centers for analysis. Additional details on the structure and function of the RISE registry are described elsewhere [12].

As of December 2018, RISE held validated data from 1113 providers in 226 practices, representing approximately 32% of the US clinical rheumatology workforce. RISE can connect to most certified EHR systems in the United States; as of 2018, the registry could map to over 30 different EHRs used by rheumatologists. EHRs largely reflect those used by community rheumatologists, as academic medical centers (and therefore large EHR vendors such as Epic and Cerner) are underrepresented in the registry.

### Study Population and Period

We analyzed data on individuals with RA seen in rheumatology practices participating in RISE between January 1, 2018, and December 31, 2018. Patients included in this study were 18 years of age or older and had ≥1 International Classification of Diseases Clinical Modification, Ninth or Tenth Revision (ICD-9-CM or ICD-10-CM) code for RA with at least 1 clinical face-to-face encounter in 2018. These inclusion criteria are based on the denominator definitions for the quality measures used to calculate the outcomes of interest (see section below).

### Outcomes

We selected two measures—routine assessment of DA and FS—for patients with RA since these are key components of a treat-to-target approach and among the newer process measures introduced in the national pay scheme for performance programs, specifically for rheumatologists. The American College of Rheumatology is currently collaborating with the National Quality Forum (NQF) to develop outcome measures on the basis of DA and FS. Thus, it is especially important to understand variations in the collection of these measures and any potential factors influencing their documentation. Assessment and documentation of DA and FS outcomes were assessed at the practice-level by calculating the performance on NQF-endorsed quality measures. Performance on each measure was defined as follows: (1) DA: percentage of patients aged 18 years and older with a diagnosis of RA, whose DA was assessed using a standardized measurement tool at 50% or more face-to-face encounters for RA during the measurement period [13]; and (2) FS: percentage of patients aged 18 years and older with a diagnosis of RA, whose FS was assessed using a standardized measurement tool at least once during the measurement period [14]. The measurement period was the 12-month period between January 1 and December 31, 2018.

### Covariates

Covariates included EHR system (NextGen, eClinicalWorks, GE Centricity, eMDs, Allscripts, Amazing Charts, Aprima, others included Lytec MD, Medent, Medisoft, Raintree System IC, MD office, Integrity, Carecloud, MedTrio, Greenway/Primesuite, iPatientCare, Prime Clinical System, MacPractice MD, IMS, SRS EHR, PrognoCIS, Cerner, Practice Fusion, DrChrono, Chart Maker Clinical, STI, American Medical Software, Athena Clinicals, Praxis EMR, RheumDocs, Greenway Intergy, Athena UniCharts, and ChartLogic) and a variety of practice and patient characteristics previously associated with measure performance. Practice characteristics included the number of providers within the practice; practice type (single-specialty group practice, solo practitioner, multi-specialty group practice, other clinical settings, and large health system); and geographical region in the United States (Northeast, Midwest, South, and West). Practice-level sociodemographic variables were calculated by aggregating the characteristics of patients included in the study and included the proportion of patients aged ≥65 years, females, non-White individuals, and of those with noncommercial insurance. Patient characteristics included age, sex, race/ethnicity, insurance type (private, Medicaid, Medicare, or other), and Charlson comorbidity index (CCI) score calculated in accordance with the Deyo modification based on codes reported at any time during the study period [15].

### Practice Documentation Workflow Survey

To learn more about potential reasons for differences in performance on DA and FS measures across EHR systems, we also assessed documentation workflows among a subset of RISE practices. A survey was disseminated electronically using a commercial survey web application to the RISE practices’ providers and administrators between November 11, 2020, and April 14, 2021. The survey included 9 questions (Multimedia Appendix 1), covering the topics of practice characteristics (3 questions), and EHR system–related factors, such as the presence of a rheumatology-specific module or dedicated structured fields for PROs, which might influence DA and FS documentation workflows (6 questions).

### Statistical Analysis

We used descriptive statistics to summarize patient and practice characteristics. Multivariable linear regression was used to examine the independent effect of EHR system on practice-level performance. We used zero-inflated models when the occurrence of zeros for practice-performance was meaningful (27.7% for DA; 40.4% for FS). These models allow for modeling of overly dispersed data. The outcome variable for the zero-inflated analyses was the count (rate) of patients in a practice, who received recommended care [16]. Zero-inflated Poisson (ZIP) and zero-inflated negative binomial (ZINB) models, all adjusted for potential confounders, were compared using Akaike’s Information Criterion (AIC), Bayesian Information Criterion (BIC) values, and log-likelihood to assess the goodness of fit. The incidence rate ratio (IRR) for each of the predictor variables in both count (rate) and logit parts of the model was reported along with 95% CIs and *P* values.

All the models (linear and zero-inflated) were adjusted for practice characteristics (including practice type, size, and geographical region) and patient case-mix (including patient age, sex, race, and insurance) since these variables have been previously shown to have a significant association with performance on rheumatology quality measures [5,17], and our goal was to isolate the impact of the EHR vendor on performance. To account for differences in case-mix, we adjusted for the aggregate characteristics of patients seen in the practice (proportion of patients aged ≥65 years, proportion of females, proportion of non-White patients, and the proportion of patients with noncommercial insurance). Missing values were included in the analyses as their own separate category without imputation. For all analyses, *P* values less than .05 were considered statistically significant.

All analyses were performed using STATA statistical software (version 16, StataCorp). This study was approved by the Western Institutional Review Board, Inc. as well as the Committee on Human Research at the University of California, San Francisco.

## Results

### Practice and Subject Characteristics

Our practice-level analysis included 314,793 individuals with RA who met the inclusion criteria across 220 practices in this study; 6 practices had no patients who met the inclusion criteria and were therefore excluded. Among all included patients (N=314,793), the mean age was 62.0 (SD 14.3) years, 76.1% were female, 67.7% were White, and 31.8% had private insurance. Most practices were single-specialty group practices (56.8%). NextGen was the most commonly used EHR system (34.1% of practices), followed by eClinicalWorks (14.5%) and Amazing Charts (9.5%) (Table 1).

**Table 1:** Characteristics of practices in the Rheumatology Informatics System for Effectiveness registry.

Practice characteristics		Practices (n=220)
**Providers in practice, n (%)**		
	1-4	162 (73.6)
	5-9	44 (20.0)
	10-20	14 (6.4)
**Practice type, n (%)**		
	Single-specialty group practice	125 (56.8)
	Solo practitioner	62 (28.2)
	Multi-specialty group practice	29 (13.2)
	Health system	4 (1.8)
**Electronic health record system, n (%)**		
	NextGen	75 (34.1)
	eClinicalWorks	32 (14.5)
	Amazing Charts	21 (9.5)
	eMDs	11 (5.0)
	GE Centricity	10 (4.5)
	Allscripts	8 (3.6)
	Aprima	8 (3.6)
	Other^a^	55 (25.0)
**US regions**		
	South	98 (44.5)
	West	48 (21.8)
	Northeast	42 (19.1)
	Midwest	32 (14.5)
**Practice-level patient characteristics, mean (SD)**		
	Proportion of patients aged ≥65 years	0.47 (0.10)
	Proportion of female patients	0.77 (0.04)
	Proportion of non-White patients	0.33 (0.26)
	Proportion with noncommercial insurance	0.68 (0.21)

^a^“Other” electronic health systems included any system used in <2% of practices, including Lytec MD, Medent, Medisoft, Raintree System IC, MD office, Integrity, Carecloud, MedTrio, Greenway/Primesuite, iPatientCare, Prime Clinical System, MacPractice MD, IMS, SRS EHR, PrognoCIS, Cerner, Practice Fusion, DrChrono, Chart Maker Clinical, STI, American Medical Software, Athena Clinicals, Praxis EMR, RheumDocs, Greenway Intergy, Athena UniCharts, and ChartLogic.

**Figure 1:**
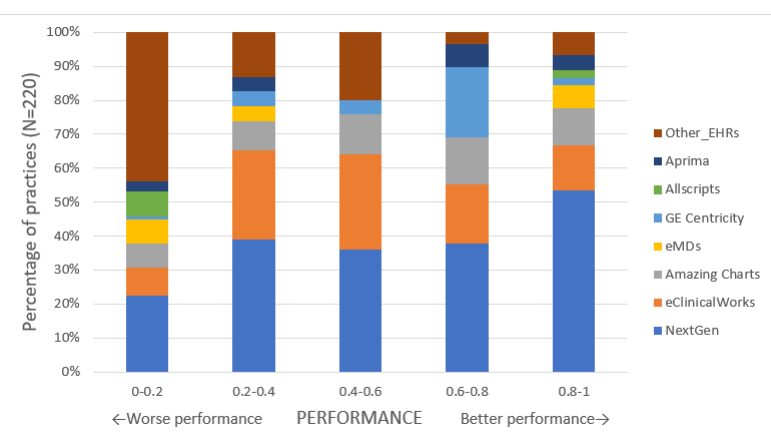
Distribution of practice-level performance on the disease activity quality measure for patients with rheumatoid arthritis, stratified by the electronic health record system.

**Figure 2:**
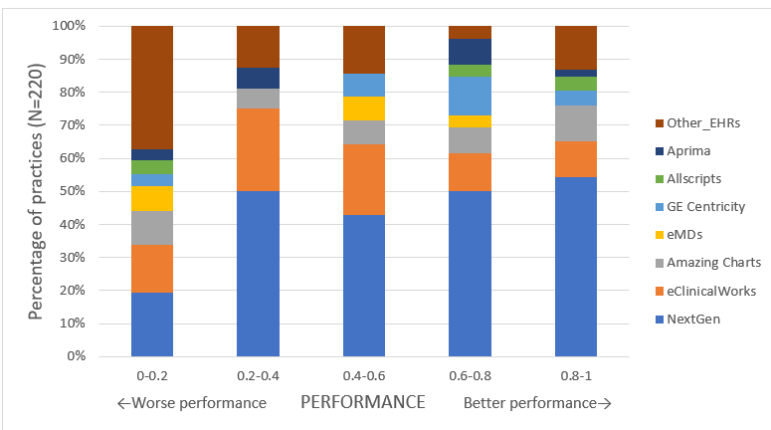
Distribution of practice-level performance on the functional status quality measure for patients with rheumatoid arthritis, stratified by the electronic health record system.

Overall, median (IQR) practice-level performance on DA and FS quality measures was 30.1 (0, 74.8) and 9.0 (0, 74.2), respectively. Figures 1 and 2 demonstrate the differences in quality measure performance on the DA and FS measures for practices using different EHR systems.

In unadjusted analyses, practices using NextGen showed significantly higher performance on DA and FS measures compared to practices using other EHR systems (Table 2). In multivariate linear regression analyses adjusting for practice characteristics and patient case-mix, practices that used NextGen had higher performance. Specifically, NextGen practices performed significantly better than Allscripts on the DA measure (51.4% vs 5.0%) and significantly better than eClinicalWorks and eMDs on the FS measure (49.3% vs 29.0% and 10.9%, respectively; Table 2). Full models with parameter estimates for practice and case-mix variables are included in Multimedia Appendix 2.

**Table 2:** Association of practice characteristics with measure performance, with marginal means estimated using multivariate regression models.

Electronic health record system	Disease activity measure performance					Functional status measure performance			
	Unadjusted performance, % (95% CI)	*P* value	Adjusted performance, % (95% CI)	*P* value	Unadjusted performance, % (95% CI)		*P* value	Adjusted performance, % (95% CI)	*P* value
NextGen	52.2 (44.5-59.9)	Reference	51.4 (42.3-60.5)	Reference	51.0 (42.9-59.2)		Reference	49.3 (39.4-59.3)	Reference
eClinicalWorks	45.7 (33.8-57.5)	.36	42.5 (30.0-54.9)	.29	30.3 (17.9-42.8)		.01	29.0 (15.4-42.6)	.03
Amazing Charts	46.7 (32.1-61.3)	.52	50.6 (35.3-65.8)	.93	32.1 (16.7-47.4)		.03	36.5 (19.8-53.2)	.22
eMDs	31.4 (11.3-51.6)	.06	29.8 (9.6-50.1)	.06	11.3 (0-32.6)		.001	10.9 (0-33.0)	.003
GE Centricity	62.0 (40.9-83.2)	.39	51.7 (30.1-73.4)	.99	48.6 (26.3-70.8)		.84	47.3 (23.8-70.8)	.88
Allscripts	11.4 (0-35.0)	<.001	5.0 (0-29.2)	.001	30.7 (5.8-55.6)		.13	28.1 (1.6-54.5)	.15
Aprima	46.1 (22.4-69.7)	.63	48.7 (24.6-72.8)	.84	33.6 (8.7-58.5)		.19	35.2 (8.9-61.5)	.34
Other^a^	13.2 (4.1-22.2)	<.001	17.3 (8.1-26.6)	<.001	15.0 (5.5-24.5)		<.001	16.8 (6.7-27.0)	<.001

^a^Other electronic health records included any system used in <2% of practices, including Lytec MD, Medent, Medisoft, Raintree System IC, MD office, Integrity, Carecloud, MedTrio, Greenway/Primesuite, iPatientCare, Prime Clinical System, MacPractice MD, IMS, SRS EHR, PrognoCIS, Cerner, Practice Fusion, DrChrono, Chart Maker Clinical, STI, American Medical Software, Athena Clinicals, Praxis EMR, RheumDocs, Greenway Intergy, Athena UniCharts, and ChartLogic. Adjusted models were adjusted for practice characteristics and patient case-mix.

Marginal means were estimated using multivariate regression models. Confidence intervals of <0 were truncated at 0.

Because we found a significant number of practices with zero performance (27.7% for DA; 40.4% for FS), we also used zero-inflated models to analyze the association between EHR systems and DA and FS documentation (Multimedia Appendix 3). Zero-inflated negative binomial models revealed that the differences in performance across EHRs were driven largely by the practices with absent documentation of DA and FS (zero performance): although we found no differences in the count portion of the ZINB model across EHRs, there were significant differences between NextGen versus other EHRs in the logit portion of the model. For example, practices using Allscripts had approximately a 2.5 times higher rate of having zero performance on DA and FS compared to practices that used NextGen (Table 3). On the other hand, practices that used GE Centricity were less likely to have zero performance on DA than practices that used NextGen (*P*<.01).

**Table 3:** Adjusted zero-inflated negative binomial models examining the main effect of electronic health record systems on the number of patients with rheumatoid arthritis who received recommended care.

Electronic health record system			Disease activity measure performance					Functional status measure performance		
			Incidence rate ratio^a^, ratio (95% CI)		*P* value		Incidence rate ratio, ratio (95% CI)			*P* value
**Count Model**										
	NextGen	Reference		Reference		Reference			Reference	
	eClinicalWorks	0.84 (0.59-1.18)		.31		0.84 (0.55-1.31)			.45	
	GE Centricity	0.88 (0.61-1.27)		.50		1.10 (0.66-1.82)			.71	
	eMDs	0.81 (0.44-1.48)		.49		0.67 (0.20-2.27)			.52	
	Allscripts	0.37 (0.07-1.98)		.25		1.08 (0.72-1.63)			.72	
	Amazing Charts	1.23 (0.78-1.92)		.37		1.34 (0.80-2.25)			.27	
	Aprima	1.16 (0.79-1.71)		.46		1.27 (0.78-2.07)			.35	
	Others^b^	0.81 (0.50-1.32)		.40		1.15 (0.78-1.69)			.49	
**Zero Inflated Model**										
	NextGen	Reference		Reference		Reference			Reference	
	eClinicalWorks	0.21 (–1.27 to 1.69)		.78		1.80 (0.72 to 2.89)			*<.001* ^c^	
	GE Centricity	–19.01 (–20.08 to –17.94)		*<.001*		1.47 (–0.11 to 3.05)			.07	
	eMDs	1.92 (0.41 to 3.43)		*.01*		2.87 (1.40 to 4.34)			*<.001*	
	Allscripts	2.48 (0.85 to 4.11)		*.003*		2.82 (1.17 to 4.48)			*<.001*	
	Amazing Charts	1.02 (–0.38 to 2.42)		.16		2.21 (1.03 to 3.40)			*<.001*	
	Aprima	1.97 (0.28 to 3.65)		*.02*		2.31 (0.70 to 3.93)			*.01*	
	Other^b^	3.17 (2.13 to 4.21)		*<.001*		3.37 (2.36 to 4.39)			*<.001*	

^a^In the count model, the incidence rate ratios represent the rate of having patients who received recommended care compared to NextGen; in the zero inflated model, the incidence rate ratios represent the rate of having zero performance compared to NextGen. Incidence rate ratios were adjusted for practice characteristics and patient case-mix.

^b^Other electronic health record systems included any system used in <2% of practices, including Lytec MD, Medent, Medisoft, Raintree System IC, MD office, Integrity, Carecloud, MedTrio, Greenway/Primesuite, iPatientCare, Prime Clinical System, MacPractice MD, IMS, SRS EHR, PrognoCIS, Cerner, Practice Fusion, DrChrono, Chart Maker Clinical, STI, American Medical Software, Athena Clinicals, Praxis EMR, RheumDocs, Greenway Intergy, Athena UniCharts, and ChartLogic.

^c^Italicized *P* values are statistically significant.

Finally, among 40 (18.2%) practices with survey responses, NextGen was the most commonly used EHR system (37.5%), followed by eClinicalWorks (22.5%) and Amazing Charts (7.5%); other EHR systems accounted for 32.5%. The majority of the responding practices using NextGen (93.3%) reported that they relied on structured data fields for DA and FS quality measure documentation in the EHR. Conversely, the vast majority of the non-NextGen practices reported that clinicians documented DA and FS in clinical notes. After the survey was closed, we additionally queried survey respondents to understand local workflows. For example, we found that NextGen includes rheumatology-specific templates that facilitate documentation of RA outcomes and functionality to track this information over time. In contrast, those using Amazing Charts enter DA and FS measures in a semistructured way (ie, in the same section of the note for every patient). This workflow, although it departs from the structured fields used by most NextGen practices, allows the registry vendor to manually extract these data for national performance reporting but is not amenable to tracking outcomes over time.

## Discussion

### Principal Findings

Although quality measures are often used to evaluate the performance of individual clinicians or health systems, the impact of health information technology on performance remains extremely understudied. We used a unique data source, the ACR’s RISE registry, which captures data from rheumatology practices across the United States, to investigate the relationship between performance on nationally endorsed RA quality measures and the EHR system used by practices.

We found that after adjusting for both practice characteristics and patient case-mix, performance in practices using some EHR systems was consistently higher; the EHRs with the highest performance generally had rheumatology-specific templates or modules in their foundation software, which facilitated collection and tracking of key RA outcomes. These findings raise important questions about the role of EHR vendors in creating software that facilitates high quality of care in rheumatology.

In both rheumatology and more general practice, studies that formally assess the impact of health information technology systems, including EHRs, on quality measure performance are limited. In a prior study using the RISE registry, we found that NextGen practices were able to improve performance on quality measures more rapidly over time than practices with other EHR systems [17]. Literature is emerging to support the notion that EHR systems can be an important factor in quality of care; for example, one study investigated a large group of primary care practices with different EHRs and their frequency of unsafe prescribing of cyclosporine, tacrolimus, and diltiazem and found important differences across EHRs [18]. In the case of RISE practices, NextGen practices reported the availability of rheumatology-specific templates in the EHR foundation software to capture key RA outcomes, facilitating quality measurement and disease-tracking. Practices using EHRs that lack this feature are much less likely to document effectively, although some of them have managed to find other methods (eg, semistructured data collection in clinical notes) for increasing accurate registry data measures for use in quality measure calculations. However, this type of documentation is less conducive to chronic disease management since it renders longitudinal tracking of disease outcomes using the EHR challenging.

Using current technology, documentation of key RA outcomes in structured EHR data fields remains the most feasible way to ensure accurate data capture and quality measurement. However, in the future, it is possible that extraction of information from clinical notes will become easier. The application of advanced approaches using text searches, natural language processing algorithms, or machine learning to extract information about disease outcomes directly from clinical notes could become more feasible in the future [19], although these strategies have largely not been demonstrated to be reliable in nonresearch settings to date and are often very cost prohibitive [20]. On the other hand, our study illustrates that is feasible in the short-term for EHR systems to modify their foundation software to include content that facilitates rheumatology practice, including capture of key RA outcomes such as DA and FS.

Further, although the idea is provocative, we think it is time for health care quality measurement to consider the range of factors that influence performance. Using a broader quality measurement paradigm, EHR vendors, in addition to individual physicians or health systems, should share in incentives or penalties associated with quality measure performance for chronic diseases such as RA [9,21,22]. Although in theory, a marketplace with multiple competitive EHR systems might have resulted in lower prices and increased functionality across all systems, in reality, the costs of switching EHR systems is high, and the risk of downtime or loss of historical data makes changing EHR vendors difficult. A shared incentive model could encourage EHR vendors to implement tools to support quality measurement and improvement. Others have explored this idea of shared responsibility for performance, particularly in the realm of patient safety [23]. For example, poorly designed user interfaces can reduce clinicians’ access to key data needed to ensure patient safety [24]. In a model designed around shared responsibility, EHR vendors would be incentivized to incorporate a user-centered process in designing and building software to support clinicians in meeting key quality metrics. Whether around patient safety or management of chronic diseases like RA, this would require EHR vendors to develop ongoing strategies to track the usability of their product and to set up systems for innovation and continuous quality improvement of software [25]. Public and private agencies could be charged with creating programs to incentivize ongoing usability-testing and to track resulting measure performance [26]. Such a model would, of course, require the full collaboration of practices and clinicians, who would need to incorporate such usability testing into their workflows and be willing to alter existing workflows to take advantage of software improvements.

### Strengths and Limitations

Our study has important strengths. Data were collected passively from different EHR systems and includes all patients with RA who met criteria for the denominator of the quality measure at each practice, thus greatly reducing the risk of selection bias inherent in other study designs. Moreover, the large number of practices and EHR systems represented in the RISE registry provides a unique view of a range of rheumatology practices using different EHR systems across the United States. Along with these strengths, this study has some important limitations. Information obtained from the EHR reflects care documented rather than care delivered. Clinicians may have assessed DA or FS for their patients but failed to document them or documented them in ways that are not easily retrievable by the registry; however, since our intention was to examine capture of data in EHRs, which are used to quality measure performance, this limitation does not impact our conclusions. A second, important limitation, is that we were limited to studying the EHRs of practices that participate in RISE. The registry currently has limited coverage of some EHRs with a greater market share among academic practices in the United States (eg, Epic and Cerner) and covers only an estimated 32% of the US clinical rheumatology workforce. Further research is needed to assess the relationships between EHR systems and quality measure performance, especially in academic settings.

### Conclusions

In summary, this study shows a strong relationship between the EHR system used by practices and performance on DA and FS quality measures for RA. Future research should investigate whether features of EHRs, which facilitate documentation and tracking of RA outcomes, facilitate improved outcomes among patients with RA over time. Developing rheumatology-specific standards across EHRs may promote routine collection of RA measures, which, in turn, could improve RA outcomes.

## Appendix

Multimedia Appendix 1 The questionnaire used in the patient-reported outcomes documentation workflow survey.

Multimedia Appendix 2 Association of practice characteristics with measure performance, using multivariate linear regression models.

Multimedia Appendix 3 Comparisons of zero-inflated Poisson and zero-inflated negative binomial models’ characteristics on count patient who received recommended care.

## Abbreviations

ACR: American College of Rheumatology
AHRQ: Agency for Healthcare Research and Quality
AIC: Akaike’s Information Criterion
BIC: Bayesian Information Criterion
CCI: Charlson comorbidity index score
CMS: Centers for Medicare & Medicaid Services
DA: disease activity
EHR: electronic health record
FS: functional status
ICD: International Classification of Diseases
IRR: Incidence rate ratio
NQF: National quality forum
PRO: patient-reported outcome
RA: rheumatoid arthritis
RISE: Rheumatology Informatics System for Effectiveness
ZINB: zero-inflated negative binomial
ZIP: zero-inflated Poisson
