# Supplementary material for: The Relationship Between Electronic Health Record System and Performance on Quality Measures in the American College of Rheumatology’s Rheumatology Informatics System for Effectiveness (RISE) Registry: Observational Study
Source: JMIR Med Inform. 2021 Nov 12;9(11):e31186. doi: 10.2196/31186 (PMC8727049; doi:10.2196/31186)
Supplement: Multimedia Appendix 1 [file medinform_v9i11e31186_app1.docx]

Appendix 1: Questionnaire used in patient reported outcomes documentation workflow survey

| Practices’ characteristics | |
| --- | --- |
|  | What is your professional setting?   - Single-specialty Group Practice - Multi-specialty Group Practice - Solo Practice - Other clinical setting - Health System |
|  | What is the number of providers in the practice?   - ≤ 4 providers - ≥ 5 ≤ 9 providers - ≥ 10 ≤ 20 providers |
|  | How many patient visits does your practice see per day?   - ≤ 50 patients - 50 ≤ 100 patients - ≥ 100 patients |
| EHR related questions | |
|  | What is the type of EHR system?   - NextGen - eClinicalWorks - Amazing Charts - eMDs - GE Centricity - Allscripts - Aprima - Others [Please specify] |
|  | Does your EHR have a specific "rheumatology module"?   - Yes - No - Don’t know |
|  | Does your EHR have a dedicated structured field (not a free text or clinical note field) that makes documenting RA disease activity possible?   - Yes - No - Don’t know |
|  | What method do you currently use for documenting disease activity on a regular basis?   - Clinical notes - Scanned document - Separate software - Other [Please specify] - Not applicable |
|  | Does your EHR have a dedicated structured field (not a free text or clinical note field) that makes documenting RA-functional status possible?   - Yes - No - Don’t know |
|  | What method do you currently use for documenting functional status on a regular basis?   - Clinical notes - Scanned document - Separate software - Other [Please specify] - Not applicable |
