# Supplementary material for: The Relationship Between Electronic Health Record System and Performance on Quality Measures in the American College of Rheumatology’s Rheumatology Informatics System for Effectiveness (RISE) Registry: Observational Study
Source: JMIR Med Inform. 2021 Nov 12;9(11):e31186. doi: 10.2196/31186 (PMC8727049; doi:10.2196/31186)
Supplement: Multimedia Appendix 2 [file medinform_v9i11e31186_app2.docx]

**Appendix 2. Association of practice characteristics with measure performance, using multivariate linear regression models.**

|  | **Disease activity measure performance** | | **Functional status measure performance** | |
| --- | --- | --- | --- | --- |
|  | coef. (95% CI) | p-value | coef. (95% CI) | p-value |
| **Practice Characteristics** |  |  |  |  |
| Provider count |  |  |  |  |
| ≤4 | ref |  | ref |  |
| 5-9 | 8.8 (-10.7, 28.2) | 0.38 | 1.0 (-20.3, 22.2) | 0.93 |
| ≥ 10 | 11.1 (-1.5, 23.8) | 0.08 | 10.6 (-3.3, 24.4) | 0.13 |
| EHR |  |  |  |  |
| NextGen | ref |  | ref |  |
| Allscripts | **-46.5 (-72.9, -20.1)** | **0.001** | -21.3 (-50.1, 7.6) | 0.15 |
| Amazing Charts | -0.9 (-19.6, 18.0) | 0.93 | -12.8 (-33.4, 7.7) | 0.22 |
| Aprima | -2.7 (-29.1, 23.7) | 0.84 | -14.1 (-43.0, 14.7) | 0.34 |
| GE Centricity | 0.2 (-23.6, 24.1) | 0.99 | -2.1 (-28.1, 23.9) | 0.88 |
| other | **-34.1 (-47.7, -20.5)** | **<0.001** | **-32.5 (-47.4, -17.6)** | **<0.001** |
| eClinicalWorks | -8.9 (-25.5, 7.6) | 0.29 | **-20.3 (-38.4, -2.2)** | **0.03** |
| eMDs | -21.6 (-44.3, 1.1) | 0.06 | **-38.5 (-63.3, -13.7)** | **0.003** |
| Region |  |  |  |  |
| Northeast | ref |  | ref |  |
| Midwest | 12.4 (-4.5, 29.3) | 0.15 | 1.03 (-17.4, 19.5) | 0.91 |
| South | 11.4 (-1.6, 24.4) | 0.08 | 4.42 (-9.7, 18.6) | 0.54 |
| West | 12.2 (-2.8, 27.2) | 0.11 | 4.75 (-11.6, 21.1) | 0.57 |
| Practice Type |  |  |  |  |
| Single Specialty Group Practice | ref |  | ref |  |
| Health System | -22.8 (-57.5, 12.0) | 0.20 | -17.2 (-55.2, 20.8) | 0.37 |
| Multi-Specialty Group Practice | 5.3 (-9.44, 20.0) | 0.48 | -5.5 (-21.5, 10.6) | 0.50 |
| Solo Practitioner | -0.3 (-11.7, 11.1) | 0.96 | -5.4 (-17.9, 7.1) | 0.39 |
| **Patient Characteristics** |  |  |  |  |
| % patients ≥ 65 | -0.4 (-0.9, 0.2) | 0.18 | 0 (-0.5, 0.6) | 0.93 |
| % non-white patients | -0.1 (-0.3, 0.1) | 0.29 | -0.1 (-0.3, 0.2) | 0.55 |
| % female patients | **-1.5 (-2.7, -0.2)** | **0.02** | 0.3 (-1.1, 1.7) | 0.66 |
| % with non-commercial insurance | -0.1 (-0.4, 0.1) | 0.33 | 0 (-0.3, 0.3) | 0.92 |

*Other included any EHR used in < 2% of practices, including Lytec MD, Medent, Medisoft, Raintree System IC, MD office, Integrity, Carecloud, MedTrio, Greenway/Primesuite, iPatientCare, Prime Clinical System, MacPractice MD, IMS, SRS EHR, PrognoCIS, Cerner, Practice Fusion, DrChrono, Chart Maker Clinical, STI, American Medical Software, Athena Clinicals, Praxis EMR, RheumDocs, Greenway Intergy, Athena UniCharts, ChartLogic. Adjusted models were adjusted for practice characteristics and patient case-mix.

Marginal means estimated using multivariate regression models.

Confidence intervals <0 were truncated at 0.
