# Supplementary material for: The Relationship Between Electronic Health Record System and Performance on Quality Measures in the American College of Rheumatology’s Rheumatology Informatics System for Effectiveness (RISE) Registry: Observational Study
Source: JMIR Med Inform. 2021 Nov 12;9(11):e31186. doi: 10.2196/31186 (PMC8727049; doi:10.2196/31186)
Supplement: Multimedia Appendix 3 [file medinform_v9i11e31186_app3.docx]

Appendix 3: Comparisons of zero-inflated Poisson (ZIP) and zero-inflated negative binomial (ZINB) models’ characteristics on count patient who received recommended care^*^

| Model characteristics | **Disease activity** | | **Functional status** | |
| --- | --- | --- | --- | --- |
|  | ZIP | ZINB | ZIP | ZINB |
| Log Likelihood | -24794.75 | -1263.622 | -24843.99 | -1083.143 |
| Akaike’s Information Criterion (AIC) | 49649.51 | 2591.244 | 49747.97 | 2230.286 |
| Bayesian Information Criterion (BIC) | 49751.32 | 2699.84 | 49849.78 | 2338.882 |

Abbreviations: ZIP: zero-inflated Poisson and ZINB: zero-inflated negative binomial ZINB

^*^Prior to considering which zero-inflated model should be used for our data analysis, we examined zero-inflated Poisson (ZIP) and zero-inflated negative binomial (ZINB) models to deal with outcome data with excessive zero counts. Zero-inflated negative binomial models were the most appropriate for the current data. This was demonstrated by the over-dispersed nature of the data (outcome data variance is higher than the mean), for disease activity: mean=709.8 (variance: 1432659), and for functional status: mean=600.2 (variance: 1275981).
